# Diagnosis of Alzheimer's disease using highly specific amyloid and tau positron emission tomography

**DOI:** 10.1002/alz.083873

**Published:** 2025-01-09

**Authors:** Hiroshi Matsuda, Haruo Hanyu, Chikako Kaneko, Tensho Yamao

**Affiliations:** ^1^ Southern TOHOKU Research Institute for Neuroscience, Koriyama Japan; ^2^ Southern TOHOKU Research Institute for Neuroscience, Tokyo Japan; ^3^ Southern Tohoku Research Institute for Neuroscience, Koriyama Japan; ^4^ Fukushima Medical University, Fukushima Japan

## Abstract

**Background:**

The Alzheimer's disease (AD) research framework has advocated defining AD as a function of the presence or absence of amyloidopathy (A), tauopathy (T), and neurodegeneration/neuronal injury (N) biomarkers, as well as the clinical syndrome. Recent advances have made it possible to perform A/T/N classification in vivo using neuroimaging techniques such as amyloid positron emission tomography (PET), tau PET, and MRI or fluorodeoxyglucose PET. Under this new framework, AD is diagnosed when both A and T are positive, regardless of whether N is positive or negative. In this study, we investigated this classification by PET using ^18^F‐NAV4694, which has a high dynamic range with low nonspecific accumulation of amyloid, and ^18^F‐MK6240, which specifically accumulates 3R/4R tau seen in AD, in patients with cognitive impairment and suspected AD.

**Method:**

Twenty‐three patients with cognitive impairment and suspected AD (12 females, 11 males, 72.1 ± 6.6 years old, Mini‐Mental State Examination; MMSE score 25.3 ± 2.1) underwent both ^18^F‐NAV4694 (300MBq) and ^18^F‐MK6240 (185MBq) PET scans using semiconductor PET/CT (United Imaging Healthcare, uMI780). Structural MRI was analyzed using VSRAD (Voxel‐based Specific Regional analysis for Alzheimer's disease ) software for voxel‐based morphometry. In addition, amyloid and tau protein deposition were quantified using the Centiloid scale and standardized uptake value ratio (SUVR) with the whole cerebellum and cerebellar cortex as reference regions, respectively.

**Result:**

Eleven patients were classified as A+T+N+ and one as A+T+N‐ and diagnosed with AD. The other classifications were A‐T‐N‐ in 5 patients, A‐T‐N+ in 2, and A+T‐N‐, A+T‐N+, A‐T+N‐, and A‐T+N+ in 1 each. The Centiloid scale of amyloid deposition did not significantly correlate with the MMSE score (r=‐0.368, p=0.083), but the composite SUVR of tau deposition did (r=‐0.463, p=0.025).

**Conclusion:**

A/T/N classification by PET and MRI correctly diagnosed AD in 12 of 23 cases (52%). Tau PET with ^18^F‐MK6240 was found to be as important in the diagnosis of AD as amyloid PET, with 2 of 14 cases (14%) being 3R/4R tau negative despite being amyloid positive. These results suggest that highly specific amyloid and tau PET can be used to accurately diagnose AD, and that tau deposition correlates well with cognitive function.

